# The Impact of Diet-Induced Weight Loss on Inflammatory Status and Hyperandrogenism in Women with Polycystic Ovarian Syndrome (PCOS)—A Systematic Review and Meta-Analysis

**DOI:** 10.3390/jcm13164934

**Published:** 2024-08-21

**Authors:** Salih Atalah Alenezi, Nusaiba Elkmeshi, Abdullah Alanazi, Sulaiman T. Alanazi, Raheela Khan, Saad Amer

**Affiliations:** 1 Division of Translational Medical Sciences, School of Medicine, University of Nottingham, Nottingham NG5 1PB, UK or salenezi@pmmc.med.sa (S.A.A.); nusaiba.elkmeshi@nottingham.ac.uk (N.E.); raheela.khan@nottingham.ac.uk (R.K.); 2 Prince Mohammed Bin Abdulaziz Medical City, Ministry of Health, Riyadh 14214, Saudi Arabia; 3 Health Sciences, Applied Sciences, Petaling Jaya 47301, Malaysia; alanzai.phdscholar@lincoln.edu.my (A.A.); alanazi.phdscholar@lincoln.edu.my (S.T.A.)

**Keywords:** PCOS, chronic inflammation, hyperandrogenism, CRP, obesity, weight loss

## Abstract

**Background:** Currently, the primary strategy for addressing polycystic ovarian syndrome (PCOS) involves lifestyle modifications, with a focus on weight loss. The purpose of this meta-analysis was to assess the impact of weight loss through dietary interventions on inflammatory status and hyperandrogenism in PCOS women. **Methods:** A comprehensive search was conducted to identify randomised controlled trials (RCTs) and cohort studies assessing the impact of diet-induced weight loss on circulating inflammatory markers (CRP, IL-6, IL-1β, TNF-α), androgens (testosterone, androstenedione), SHBG, and luteinising hormone (LH) in PCOS women. The quality and risk of bias of the included studies were assessed using the Cochrane Collaboration’s tool for RCTs and the Newcastle–Ottawa Scale for cohort studies. Data were entered into RevMan software v5.9 for the calculation of standard mean difference (SMD) and the 95% confidence interval (95%CI) of circulating inflammatory markers, androgens, and LH between baseline and post-weight loss values. **Results:** Eleven studies (*n* = 323) were eligible for the systematic review, of which nine (*n* = 286) were included in the meta-analysis. Pooled analysis of data revealed a statistically significant decrease in circulating CRP (SMD 0.39, 95%CI 0.22, 0.56; 9 studies, *n* = 286), IL-6 (SMD 0.37, 95%Cl, 0.12, 0.61; 3 Studies, *n* = 140), TNF-α (SMD 0.30, 95%Cl, 0.07, 0.53; 4 Studies, *n* = 162), androstenedione (SMD 0.36, 95%Cl, 0.13, 0.60; 4 studies, *n* = 147) and LH (SMD 0.30, 95% Cl, 0.09, 0.51; 5 studies, *n* = 197) after weight loss compared to baseline levels among PCOS women. A meta-analysis of five studies (*n* = 173) showed a statistically significant increase in circulating SHBG after weight loss compared to baseline levels (SMD −0.43, 95%Cl, −0.65, −0.21). **Conclusions:** These findings suggest that weight loss induced by dietary interventions seems to improve PCOS-related chronic inflammation and hyperandrogenism. The possible causative relationship between the improvement in inflammation and hyperandrogenism remains to be determined.

## 1. Introduction

Polycystic ovary syndrome (PCOS) is a common and diverse disorder that affects reproductive, endocrine, and metabolic functions in women [1]. The prevalence of PCOS among women of reproductive age varies, ranging from 6% to 25% depending on the diagnostic criteria used [2]. A significant proportion of women with PCOS are overweight or obese, with rates reported up to 61% [3], and a substantial number also experience insulin resistance, ranging from 44% to 70% [4]. Insulin resistance is a central factor in the development of hyperandrogenism and chronic inflammation in women with PCOS [5]. Elevated levels of inflammatory markers such as C-reactive protein (CRP) are associated with an increased risk of cardiovascular disease, metabolic syndrome, and type 2 diabetes mellitus (T2DM) in women with PCOS [6].

CRP is produced by the liver in response to IL-6 and TNF-α [7], and is considered both an indicator of low-grade chronic inflammation and an active contributor to the development of atherosclerosis [8]. Adipose tissue releases various bioactive substances known as adipocytokines, including leptin, TNF-α, IL-6, IL-18, plasminogen activator inhibitor type 1, and adiponectin, which further contribute to inflammation and metabolic disorders [9,10]. Adiponectin is thought to have insulin-sensitising [9], antiatherogenic, and anti-inflammatory properties [11], and there is a reverse correlation between adiponectin and CRP levels [12]. Emerging evidence suggests that novel cardiovascular risk factors are also deregulated in PCOS, with increased CRP [13,14], IL-6 [15,16], and TNF-α levels [17], as well as reduced adiponectin levels observed in both obese and non-obese women with PCOS [18,19].

Hormonal manifestations in PCOS involve increased serum concentrations of androgens including testosterone, DHEAS, and androstenedione [20], and reduced sex hormone-binding globulin (SHBG) levels [21]. Most women with PCOS show elevated luteinising hormone (LH) and decreased follicle-stimulating hormone levels during the follicular phase [22], contributing to increased androgen concentrations, follicular arrest, and an accumulation of small follicles within the ovary [23].

The initial approach to treating PCOS involves lifestyle modifications, with an emphasis on weight loss, a key recommendation from the World Health Organization in the management of this condition [24]. Weight management is also recommended for infertile PCOS women undergoing assisted reproductive technology procedures [25]. Lifestyle management leads to enhancements in the reproductive, metabolic, endocrine, and psychological aspects of PCOS [26]. In the broader population, standard guidelines for weight control within lifestyle interventions include a diet that is low in fat (around 30% of total energy intake, with approximately 10% coming from saturated fat and less than 300 mg of daily cholesterol), moderate in protein (about 15%), high in carbohydrates (approximately 55%), and rich in fibre, in conjunction with regular moderate exercise [27,28]. Furthermore, previous research has indicated that adopting a nutritious eating pattern could be linked to the metabolic characteristics and levels of inflammatory cytokines in conditions associated with metabolic syndrome [29,30].

The effect of diet-induced weight loss on CRP and other inflammatory markers in PCOS women has previously been investigated in several studies with conflicting results. While some studies have reported no differences in CRP levels before and after weight loss in PCOS women [31,32], others have found that weight loss led to a reduction in CRP levels in this population [33,34]. There have been no previous systematic reviews assessing weight loss through dietary interventions alone on CRP and other inflammatory markers in PCOS women. A systematic review published in 2013 reported that weight loss through dietary intervention resulted in subtle and inconsistent improvements in anthropometric measurements, reproductive health, metabolic factors, and overall quality of life in conjunction with reductions in glycaemic load [35]. However, it remains unclear whether weight loss has a discernible effect on inflammatory markers in women with PCOS. The purpose of the current meta-analysis was therefore to conduct a comprehensive systematic review of available human studies assessing the effect of weight loss through dietary interventions on inflammatory status and hyperandrogenism in PCOS women.

## 2. Materials and Methods

This systematic review was prospectively registered with PROSPERO (registration number CRD42023412566) and carried out in accordance with the Preferred Reporting Items for Systematic Reviews and Meta-Analyses (PRISMA) criteria.

### 2.1. Eligibility Criteria for Study Selection

We considered all studies including clinical trials and cohort studies comparing the serum levels of CRP, other inflammatory markers (e.g., IL-6, IL-1β, TNF-α), androgens and LH in women with PCOS undergoing weight loss through dietary interventions. Only studies that matched and/or adjusted for age and BMI and measured circulating CRP, TNF, IL-6 and/or testosterone were included. The review only included English-language human studies with drug-naïve, nonpregnant women aged 16–39 years who had no prior medical history of conditions that could have affected their inflammation markers or reproductive endocrine profile.

### 2.2. Outcome Measures

#### 2.2.1. Primary Outcomes

Serum concentrations of CRP, IL-6, TNF-α, and/or IL-1β in PCOS women before and after weight loss through dietary intervention.

#### 2.2.2. Secondary Outcome Measures

Serum levels of the anti-inflammatory factor adiponectin, androgens (SHBG, testosterone, DHEAS, androstenedione) and LH in PCOS women before and after diet-induced weight loss. 

### 2.3. Search Strategy

EMBASE (Ovid); Medline (Ovid); CENTRAL (www.thecochranelibrary.com) accessed on 23 October 2023; Clinicaltrials.gov; the EU Clinical Trials Register; PubMed; and the World Health Organisation International Clinical Trials Register were systematically searched starting from 1946 to October 2023 for relevant studies. A combination of the following search terms was used: “Weight Loss” OR “Weight management” OR “diets intake” OR “Energy Intake” OR “LOW-CARB DIET” OR “Caloric Restriction” AND “Polycystic Ovary Syndrome” AND “inflammatory markers” OR “C-reactive protein” OR “Interleukin-6” OR “TNF-ALPHA” OR “IL-1BETA” OR “Adiponectin”. The keywords were combined using Boolean operators for each database, as appropriate.

### 2.4. Screening and Selection of Retrieved Studies

The titles and abstracts of studies retrieved by the electronic search were independently screened by two authors (SAA and AA) for relevance. Full texts of the pertinent articles were further evaluated, and eligible studies were selected according to the inclusion and exclusion criteria. A third author (STA) adjudicated any discrepancies between authors.

### 2.5. Assessment of Quality and Risk of Bias

The Cochrane Collaboration’s tool [36] was used to evaluate the quality of the included randomised controlled trials (RCTs) (Table 1). The generation of random sequences, allocation concealment, blinding of outcome assessments, incomplete outcome data, selective reporting, and other biases were among the methodological domains that were taken into consideration. However, blinding of participants could not be achieved in our research, as individuals undergoing a diet intervention cannot be effectively blinded. Each assessed item received one of three scores: low, unclear, or high for bias. For this evaluation, we referred to the quality assessment table found in the Cochrane Handbook of Systematic Reviews of Interventions [36]. The quality and risk of bias of the observational studies were evaluated using the Newcastle–Ottawa scale (NOS) for the assessment of cohort studies, based on the recommendation of the Cochrane Collaboration [36,37]. The original Newcastle–Ottawa scale for nonrandomised studies assesses three main categories, including selection, comparability, and outcomes, giving a maximum of four, two, and three stars for each category, respectively [37]. This scale was modified to suit the nature of this study giving a maximum of three stars for selection (representativeness of the exposed cohort, ascertainment of exposure, and demonstration that outcome of interest was not present at the start of the study), four for comparability (studies including PCOS women with age ≤ 40 yr, BMI > 25, using low caloric diet interventions, and studies employing the Rotterdam criteria for the diagnosis of PCOS) and two for outcome criteria (assessment of outcome and follow-up long enough for outcomes to occur) [38,39] (Table 2). Studies with six or more stars were classified as being of good quality [40]. The quality assessment was conducted by two authors (S.A.A. and A.A.), and every disagreement was resolved by a third reviewer (N.E.).

### 2.6. Data Extraction and Analysis

Data (mean ± SD) were extracted from the individual articles including demographics (age and BMI), inflammatory markers (CRP, TNF-α, IL-6, IL-1β), adiponectin, androgens (testosterone, SHBG, DHEAS, androstenedione) and LH. These data were uploaded into RevMan software, version 5.9 (The Nordic Cochrane Centre, Copenhagen, Denmark; The Cochrane Collaboration, 2011) for meta-analysis. The standardised mean difference (SMD) between baseline and post-weight loss data and 95% confidence interval (CI) were calculated for inflammatory markers and hormones. For RCTs, we only included baseline and post-weight loss data from the arm including PCOS women undergoing the dietary intervention. The SDM model was used in this meta-analysis due to the differences in CRP measurements among the included studies [41]. The SMD has been shown to be more generalisable and an easier way to assess the degree of variation between groups, in addition to being independent of the unit of measurement [42]. According to the general rule described by Cohen et al., a difference that is considered “small” is represented by an SMD of 0.2, “medium” by an SMD of 0.5, and “large” by an SMD of 0.8 [43].

To assess the statistical heterogeneity between studies, the chi-square test and I-squared (I^2^) statistics were utilised. I^2^ ≥ 50% or chi-square analyses higher than its degree of freedom indicated high heterogeneity. An initial overall meta-analysis for CRP was performed for all included investigations. Further subgrouping analysis of CRP data was conducted with a diet period more or less than 8 weeks.

## 3. Results

### 3.1. Search Results

**Figure 1:**
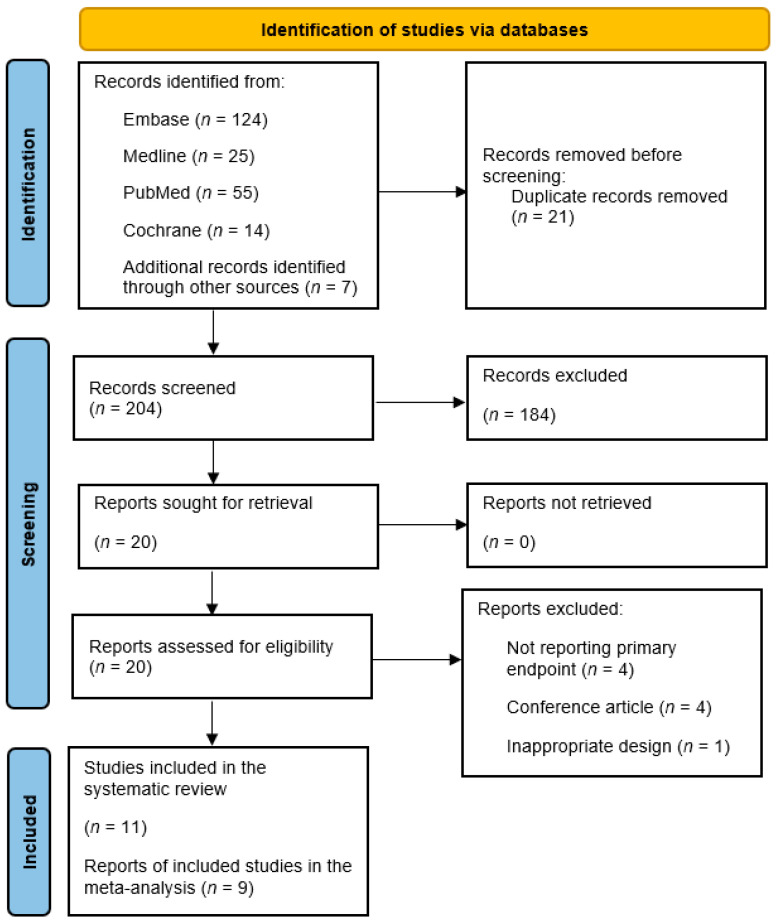
PRISMA flow chart.

The initial electronic database search identified 225 articles, which were reduced to 204 after removing duplicates. During the screening of the title and abstract, 184 irrelevant articles were excluded. After a thorough review of the full text of the remaining 20 papers, an additional 9 did not meet the eligibility criteria and were consequently excluded as illustrated in Figure 1. The remaining 11 studies fulfilled the eligibility criteria and were included in this review [26,31,32,33,34,44,45,46,47,48,49].

**Table 1:** The Newcastle–Ottawa Scale (NOS) [37] was used for assessing the cohort studies.

1st Author, Year	Selection	Comparability	Outcome	Overall
Szczuko, 2018 [45]	**	***	**	7
Moran, 2007 [49]	***	****	**	9
Asemi, 2015 [46]	**	**	**	6
Olszanecka-Glinianowicz, 2008 [48]	***	**	**	7

The star scoring system was redistributed to have a maximum of three stars for selection, four stars for comparability, and two stars for outcome criteria.

**Table 2:** Characteristics of the 11 included studies investigating changes in circulating inflammatory markers and androgens in PCOS women undergoing diet-induced weight loss.

First Author, Year	Country	Study Design	*n*	Age (Y)	Diet Intervention		BMI (Kg/m^2^)-[Weight (Kg)]			Outcomes Measured	
					Type	Duration [Weeks]	Before	After	*p*	Inflammatory Markers	Androgens
Moran, 2006 [26]	Australia	RCT	34	32.1 ± 5.2	energy-restricted diet	8	34.9 ± 7.0 [96.0 ± 3.3]	- [90.3 ± 3.3]	-	CRP	-
Mehrabani, 2012 [31]	Iran	RCT	26	28.5 ± 5.2	low-calorie diet	12	31.1 ± 4.6 [78.9 ± 12.4]	- [74.8 ± 0.5]	-	CRP, TNF-α, IL-6	SHBG
Cheshmeh, 2021 [32]	Iran	RCT	99	33.8 ± 5.4	low-calorie diet	16	35.18 ± 5.16	32.86 ± 5.95	<0.001	CRP, TNF-α, IL-6	Testosterone, SHBG, DHEAS, Androstenedione
Marsh, 2010 [33]	Australia	RCT	50	29.3 ± 0.8	low-glycaemic index diet	Up to 48	34.7 ± 0.9	33.2 ± 0.6	-	CRP	Testosterone, SHBG,
Esfahanian, 2012 [34]	Iran	RCT	13	20.0 ± 4.6	low-calorie diet	12	34.1 ± 5.4	30.1 ± 5.5	<0.001	CRP	Testosterone, DHEAS
Deshmukh, 2023 [44]	UK	RCT	11	27.7 ± 3.8	Very low-calorie diet	8	37.8 ± 3.9	33.7 ± 3.9	<0.0001	CRP	Testosterone, SHBG, DHEAS, Androstenedione
Szczuko, 2018 [45]	Poland	Cohort	22	26.6 ± 4.2	low-glycaemic index diet	12	28.38 [79.13 ± 14.58]	26.1 [73.01 ± 10.18]	<0.05	TNF-α	Testosterone, SHBG, DHEAS, Androstenedione
Asemi, 2015 [46]	Iran	Cohort	27	27.5 ± 3.6	Fasting [16.5/day]	4	28.6 ± 3.9	28.4 ± 3.9	0.64	CRP	-
Moran, 2010 [47]	Australia	RCT	14	32.8 ± 4.5	energy-restricted diet	16	37.6 ± 7.1	34.9 ± 6.5	-	CRP	-
Olszanecka-Glinianowicz, 2008 [48]	Poland	Cohort	15	28.5 ± 7.7	low-calorie diet	ND	36.1 ± 6.6	31.6 ± 5.8	<0.00001	TNF-α, IL-6	Testosterone, SHBG, DHEAS, Androstenedione
Moran, 2007 [49]	Australia	Cohort	12	31.7 ± 6.2	Energy-restricted diet	8	35.7 ± 5.8 [95.1 ± 19.3]	- [91.2 ± 15.7]	-	CRP	-

### 3.2. Risk of Bias and Quality Assessment of Selected Studies

**Figure 2:**
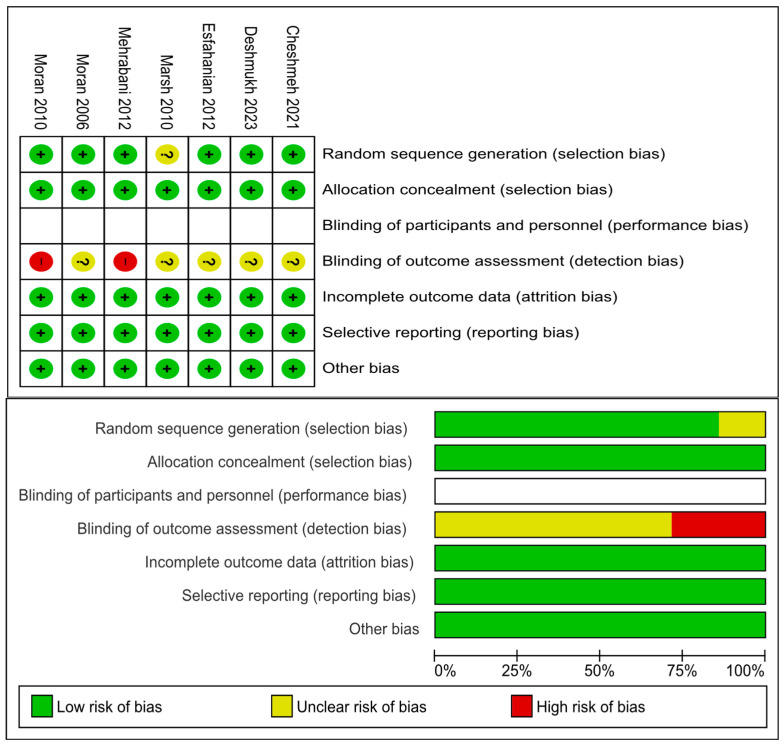
Risk of bias assessments of individual RCTs [26,31,32,33,34,44,47].

The details of risks of bias assessments within the seven included RCTs are presented in Figure 2. Six RCTs reported adequate methods of random sequence generation while the remaining RCT [44] was not clear on this. All seven RCTs reported adequate methods of allocation concealment. None of the seven RCTs blinded their participants or personnel due to the nature of diet intervention. Given that all outcomes of interest were objective outcomes, it is unlikely that non-blinding would introduce any bias. All seven RCTs had low risk of attrition and reporting bias.

Table 1 summarises the quality scores of the four cohort studies included in the review. All studies scored between 6 and 9 on the modified Newcastle–Ottawa scale.

### 3.3. Included Studies

The review included 11 eligible studies (*n* = 323) investigating changes in circulating inflammatory markers and androgens after diet-induced weight loss in women with PCOS.

#### 3.3.1. Study Designs 

A total of 7 of the 11 included studies (*n* = 247) were RCTs, while 4 (*n* = 76) were cohort studies. For the RCTs, only the weight loss intervention arm was included in the meta-analysis.

#### 3.3.2. Study Characteristics 

The characteristics of all the studies are summarised in Table 2. In 10 studies, PCOS participants underwent a hypocaloric diet to reduce weight, while the remaining study utilised a 16.5 h fasting diet style. 

#### 3.3.3. Study Participants 

A total of 323 overweight/obese women with PCOS were enrolled in all 11 included studies. An appropriate participants’ selection was used in all articles, which met our inclusion criteria. The diagnosis of PCOS in all 11 studies adhered to the Rotterdam ESHRE/ASRM criteria. Participants across these studies were within the childbearing age range of 15 to 38 years, had no endocrinological diseases, were not pregnant at the time of participation, and had not been on any medication that could impact the levels of inflammatory markers in the preceding three months.

### 3.4. Outcome Data

Of the 11 included studies, CRP was reported in 9 [26,31,32,33,34,44,46,47,49], TNF-α in 4 [31,32,45,48], IL-6 in 3 [31,32,48], and adiponectin in 1 study [31] (Table 2). However, only CRP, IL-6 and TNF-α reported data suitable for the meta-analysis. There was no sufficient data to include in a meta-analysis for adiponectin or IL-1β.

Among the 11 articles, SHBG was in 6 (*n* = 223) [31,32,33,44,45,48], DHEAS was in 6 [31,32,34,44,45,48], testosterone was reported in 6 [32,33,34,44,45,48] and androstenedione was in 4 studies (*n* = 147) [32,44,45,48] (Table 3). LH was reported in five studies (*n* = 197) [32,33,44,45,48] (Table 2).

### 3.5. Systematic Review

#### 3.5.1. Inflammatory Markers

Serum CRP levels were measured before and after diet-induced weight loss in PCOS women in nine studies, *n* = 286, of which three (*n* = 61) reported a significant decrease [26,34,47], while the remaining six (*n* = 225) showed no statistically significant change in circulating CRP after the weight loss [31,32,33,44,46,49] (Table 4).

Circulating IL-6 levels were compared before and after diet-induced weight loss in individuals with PCOS in three studies (*n* = 140), with no significant difference reported [31,32,48] (Table 3).

Serum TNF-α concentrations were measured in four studies (*n* = 162), of which three (*n* = 136) showed no change [32,45,48], while one (*n* = 26) reported a significant decrease after the diet-induced weight loss [31] (Table 3).

#### 3.5.2. Anti-Inflammatory Marker

One study (*n* = 26) assessed circulating adiponectin showing significantly increased levels after diet-induced weight loss compared to baseline levels [31] (Table 3).

#### 3.5.3. Androgens and LH

Two studies (*n* = 35) reported a significant reduction in serum testosterone levels in PCOS women after diet-induced weight loss [34,45], while four studies (*n* = 175) showed no significant change compared to baseline levels before the dietary interventions [32,33,44,45,48] (Table 4).

Two studies (*n* = 37) measuring SHBG showed a significant increase after diet-induced weight loss in PCOS women [31,44], while four studies (*n* = 186) showed no significant change compared to baseline levels before the dietary interventions [32,33,45,48] (Table 4).

Circulating androstenedione was evaluated before and after diet-induced weight loss in four studies (*n* = 147), with two (*n* = 121) reporting a significant reduction [32,45] and two (*n* = 26) showing no significant change after the weight loss compared to baseline levels [44,48] (Table 4).

Six articles (*n* = 186) assessed circulating DHEAS levels before and after diet-induced weight loss. Five studies (*n* = 160) reported no change [32,34,44,45,48], while one study (*n* = 26) showed a significant increase in post-weight loss serum DHEAS levels compared to baseline levels before dietary intervention [31] (Table 4).

Circulating LH was measured in five studies (*n* = 197), with four (*n* = 175) reporting no significant difference [32,33,44,48] and one (*n* = 22) showing no significant change after the weight loss compared to baseline levels [45] (Table 4).

### 3.6. Meta-Analysis

#### 3.6.1. Inflammatory Markers

**Figure 3:**
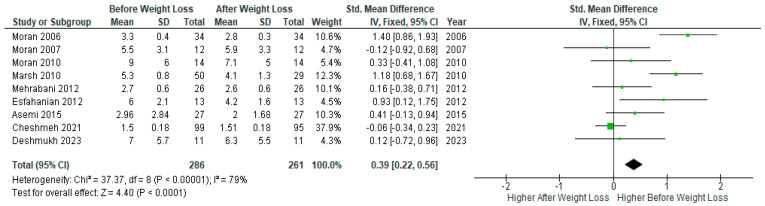
Overall CRP pooled analysis of 9 studies [26,31,32,33,34,44,46,47,49].

A pooled analysis of nine studies (*n* = 286) showed significantly lower serum CRP levels after diet-induced weight loss compared to baseline levels before weight loss in PCOS women (SMD 0.39, 95% Cl, 0.22, 0.56; z = 4.40; *p* < 0.0001; I^2^ = 79%). The heterogeneity between studies was high (Figure 3).

**Figure 4:**
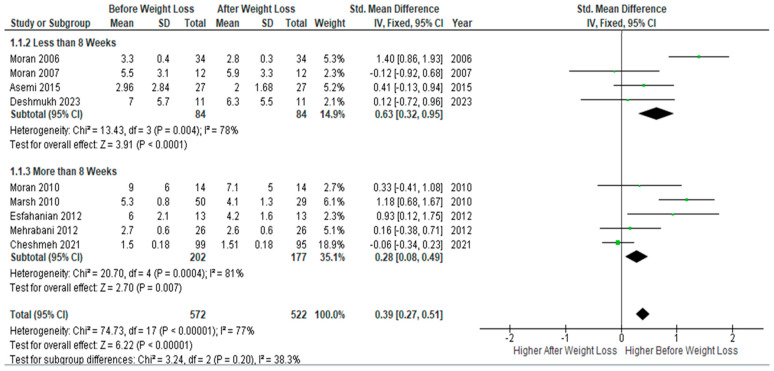
CRP pooled analysis for periods of both less than and more than eight weeks [26,31,32,33,34,44,46,47,49].

Subgroup meta-analysis of four studies (*n* = 84) with follow-up periods of ≤ 8 weeks of dietary interventions showed a statically significant decrease in serum CRP levels after weight loss compared to baseline levels (SMD 0.63, 95% Cl, 0.32, 0.95; z = 3.91; *p* < 0.0001; I^2^ = 78%). Similarly, but to a less extent, pooled analysis of five studies (*n* = 177) with follow-up periods > 8 weeks showed a significant drop in circulating CRP at follow-up assessment, but with a smaller SMD (SMD 0.28, 95% Cl, 0.08, 0.49; z = 2.70; *p* = 0.007; I^2^ = 81%). Heterogeneity between studies was low (Figure 4).

**Figure 5:**
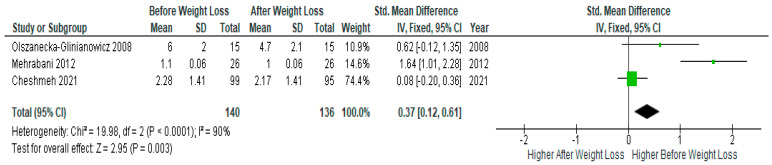
IL-6 meta-analysis of 3 studies [31,32,48].

Three studies presented IL-6 data suitable for meta-analysis (*n* = 140) and showed significantly lower serum IL-6 levels after diet-induced weight loss compared to baseline levels before weight loss in PCOS women (SMD 0.37, 95% Cl, 0.12, 0.61; z = 2.95; *p* =0.003; I^2^ = 90%). The heterogeneity between studies was high (Figure 5).

**Figure 6:**
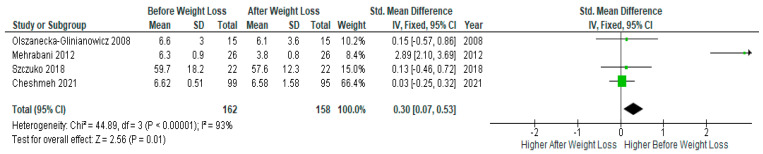
TNF-α pooled analysis of 4 studies [31,32,45,48].

Pooled data of four studies (*n* = 162) revealed significantly lower serum TNF-α levels after diet-induced weight loss compared to baseline levels before weight loss in PCOS women (SMD 0.30, 95% Cl, 0.07, 0.53; z = 2.56; *p* = 0.01; I^2^ = 93%). The heterogeneity between studies was high (Figure 6).

#### 3.6.2. Androgens and LH

**Figure 7:**
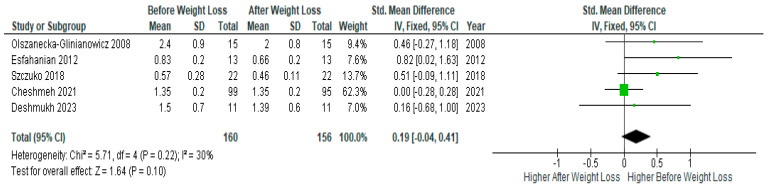
Testosterone meta-analysis of 5 studies [32,34,44,45,48].

Pooled analysis of five studies (*n* = 160) with relevant data showed no significant difference in serum testosterone levels before and after diet-induced weight loss in PCOS women (SMD 0.19, 95% Cl, −0.04, 0.41; z = 1.64; *p* = 0.10; I^2^ = 30%). Heterogeneity between studies was moderate (Figure 7).

**Figure 8:**
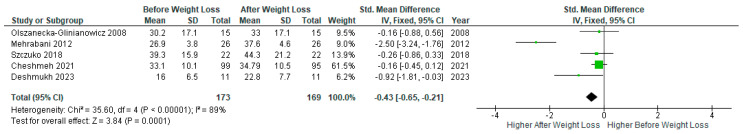
SHBG pooled analysis of 5 studies [31,32,44,45,48].

A meta-analysis of five studies (*n* = 173) with relevant data showed a significant increase in SHBG levels after diet-induced weight loss compared to baseline levels in PCOS women (SMD −0.43, 95% Cl, −0.65, −0.21; z = 3.84; *p* = 0.0001; I^2^ = 89%). The heterogeneity between studies was high (Figure 8).

**Figure 9:**
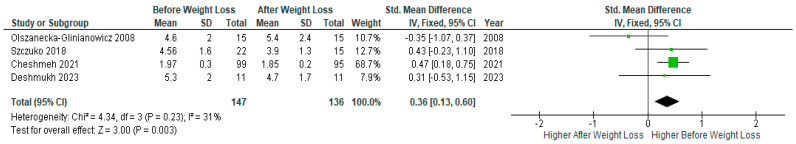
Androstenedione pooled analysis of 4 studies [32,44,45,48].

A meta-analysis of four studies (*n* = 147) with relevant data showed a statistically significant decrease in androstenedione levels after weight loss compared to baseline levels before the diet intervention in PCOS women (SMD 0.36, 95% Cl, 0.13, 0.60; z = 3.00; *p* = 0.003; I^2^ = 31%). Heterogeneity between studies was low (Figure 9).

**Figure 10:**
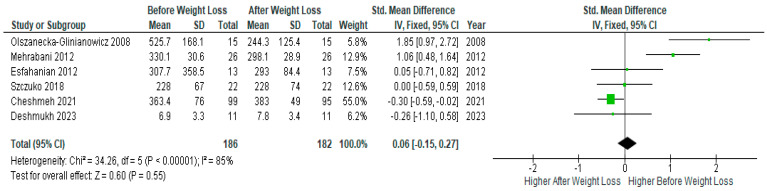
DHEAS data analysis of 6 studies [31,32,34,44,45,48].

Pooled analysis of six studies (*n* = 186) with relevant data showed no significant difference before and after diet-induced weight loss (SMD 0.06, 95% Cl, −0.15, 0.27; z = 0.60 *p* = 0.55; I^2^ = 85%). The heterogeneity between studies was high (Figure 10).

**Figure 11:**
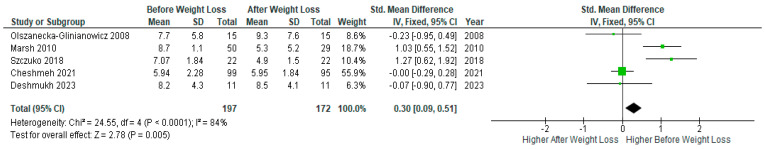
LH pooled analysis of 5 studies [32,33,44,45,48].

Meta-analysis of five studies (*n* = 197) showed a significant decrease in serum LH levels after weight loss compared to baseline levels before the diet intervention in PCOS women (SMD 0.30, 95% Cl, 0.09, 0.51; z = 2.78; *p* = 0.005; I^2^ = 84%). Heterogeneity between studies was high (Figure 11).

## 4. Discussion

To the best of our knowledge, this is the first meta-analysis that investigates the effect of weight loss through dietary intervention alone on circulating levels of inflammatory markers and reproductive endocrine hormones in PCOS women. A total of 11 studies (*n* = 298) were included in the systematic review, of which 9 (*n* = 261) were included in the meta-analysis, showing a significant reduction in serum CRP after diet-induced weight loss in PCOS women. Also, CRP sub-analysis according to the period of weight loss (≤8 weeks or >8 weeks) showed decreased levels of serum CRP in PCOS women. Also, data analysis of serum IL-6 (three studies) and TNF-α (four studies) showed a significant reduction after diet-induced weight loss in women with PCOS.

In addition, this meta-analysis evaluated the reproductive endocrine profile before and after diet-induced weight loss. Most included studies demonstrated decreased levels of androstenedione (*p* = 0.003) and LH (*p* = 0.0001) and increased levels of SHBG (*p* < 0.0001) after diet-induced weight loss in women with PCOS. However, the meta-results did not demonstrate a statistically significant reduction in serum testosterone or DHEAS levels after diet-induced weight loss; although there was a trend toward lower post-weight decrease in testosterone levels, which did not reach statistical significance, possibly due to the limited sample size.

The results of our meta-analysis are consistent with several previous studies reporting a decline in inflammatory markers after weight loss through dietary interventions. These studies have demonstrated that a reduction in BMI could result in decreased levels of serum CRP and other inflammatory markers [32,34,44,47]. Additionally, an early study investigating the association between overweight/obesity and low-grade systemic inflammation, as measured by serum CRP levels, found a correlation between higher BMI and elevated CRP concentrations [52]. Our research group provide strong evidence for a moderate increase in circulating CRP in PCOS women [53].

Our results are consistent with a previous systematic review published in 2022 by Moori et al., which investigated the effects of exercise-induced weight loss on circulating CRP and other inflammatory markers. This review reported that exercise could significantly lower serum CRP levels [54]. Our results are also in agreement with a systematic review published in 2014 that assessed the effectiveness of lifestyle interventions including exercise with or without diet intervention on the endocrine profile of PCOS individuals [55].

While the beneficial effects of weight loss on PCOS-related hyperandrogenism are well documented in the literature, our review provides a comprehensive and updated analysis of recent evidence. Our updated data highlight not only the effectiveness of diet-induced weight loss in reducing circulating androgens, but also the possible correlation between the reduction in androgens and the inflammatory markers. This elucidates the possible interplay between inflammatory markers and hyperandrogenism, although the causative nature of this correlation remains to be determined.

The main limitations of this meta-analysis are the small size of many of the included studies and the high heterogeneity between studies. The main heterogeneity is due to the wide variation in the duration and the type of diet used in different studies including low calorie, very low calorie, periodic fasting, and energy-restricted diet. Another limitation is the insufficient data for adiponectin, leading to its inclusion in the systematic review only but not rather in the meta-analysis. Additionally, it is important to acknowledge that one of the criteria in the Cochrane Risk of Bias questionnaire, regarding the blinding of participants, could not be fulfilled in our review as blinding was not possible for the included trials due to the nature of the intervention.

Possible mechanisms explaining the impact of weight loss through dietary intervention on inflammation involve the reduction in adipose tissue, resulting in decreased production of inflammatory substances [56]. Another possible mechanism is through elimination of insulin resistance, which is strongly implicated in chronic inflammation [57]. Additionally, weight loss could increase the production of adipokine, which has anti-inflammatory effects [58]. Furthermore, a healthier diet may result in a healthier gut microbiota that may in turn reduce systemic inflammation through improved gut barrier function and reduced endotoxin levels. [59,60]. Additionally, following a healthy diet could reduce oxidative stress and lower inflammatory markers [61,62].

Interestingly, this meta-analysis presents possible evidence indicating a positive association between chronic inflammation and reproductive endocrine hormones (androstenediones, and LH) in PCOS women. However, the causative nature of this association remains to be evaluated through further research.

Given the above-mentioned limitations of this review, further adequately designed and sufficiently powered studies are needed to further assess the impact of diet-induced weight loss on PCOS-related chronic inflammation and hyperandrogenism. Future studies should have robust study designs, such as randomised controlled trials with well-defined control groups, and with sufficiently calculated sample size to ensure sufficient statistical power. This will help to detect meaningful differences in outcomes related to inflammatory markers, hyperandrogenism, and other relevant parameters in women with PCOS. These investigations should also include PCOS women with both high and normal BMI levels to elucidate the effects of weight loss interventions and determine the optimal percentage reduction in BMI that has a more significant impact on their inflammatory markers and endocrine hormones. In addition, future research could also explore the possible effect of weight loss intervention on emerging inflammatory pathways including the NLRP3 inflammasome and its related components (such as Casp-1, ASC, and IL-1β) in PCOS patients.

Weight loss interventions present a range of positive outcomes for women with PCOS, with minimal side effects. This lifestyle adjustment holds promise in enhancing metabolic health by addressing insulin resistance and metabolic disruptions. Moreover, it shows potential for positively influencing fertility through beneficial changes in reproductive endocrine hormones. Furthermore, the decrease in androgens highlight the potential role of dietary interventions in improving various PCOS-related symptoms like hirsutism, acne, and menstrual irregularities. Therefore, the results of this meta-analysis underscore the importance of incorporating weight management strategies into the standard care for PCOS women. Clinicians might consider using inflammatory biomarkers as tools to monitor the effectiveness of dietary interventions, tailoring treatment plans to achieve optimal outcomes in PCOS management.

While exercise or medical interventions may not be possible or appropriate in some cases of PCOS and PCOS-related morbidities, weight loss through dietary intervention remains a suitable and effective choice.

## 5. Conclusions

The findings of this meta-analysis suggest that diet-induced weight loss seems to have a positive impact on PCOS-related chronic inflammation and hyperandrogenism. Whether there is any mechanistic correlation between the improvement in inflammation and hyperandrogenism remains to be further investigated. Given the limited number and the small size of the reviewed studies, the results of this review should be interpreted with caution. Further research with a larger sample size and adequate design is needed to further validate the evidence generated in the review.

## Figures and Tables

**Table 3:** Changes in circulating CRP and other inflammatory markers after diet-induced weight loss in PCOS women.

First Author, Year	*n*	CRP			IL-6 (pg/mL)			TNF-α (pg/mL)			Adiponectin (ng/mL)		
		Before	After	*p*	Before	After	*p*	Before	After	*p*	Before	After	*p*
Moran, 2006 [26]	34	3.30 ± 0.40 *	2.80 ± 0.30 *	<0.05	-	-	-	-	-	-	-	-	-
Mehrabani, 2012 [31]	26	2.70 ± 0.60 **	2.6 ± 00.60 **	-	1.1 ± 0.06	1.0 ± 0.06	-	6.3 ± 0.9	3.8 ± 0.8	<0.005	59.6 ± 4.4	67.7 ± 4.6	<0.005
Cheshmeh, 2021 [32]	99	1.50 ± 0.18 *	1.50 ± 0.18 *	0.1	2.28 ± 1.41	2.17 ± 1.41	0.76	6.62 ± 0.51	6.58 ± 1.58	0.45	-	-	-
Marsh, 2010 [33]	50	5.30 ± 0.80 **	4.10 ± 0.13 ** ◊◊	-	-	-	-	-	-	-	-	-	-
Esfahanian, 2012 [34]	13	60.00 ± 21.00 **	42.00 ± 16.00 **	0.04	-	-	-	-	-	-	-	-	-
Deshmukh, 2023 [44]	11	7.00 ± 5.70 **	6.30 ± 5.50 **	0.7	-	-	-	-	-	-	-	-	-
Szczuko, 2018 [45]	22	-	-	-	-	-	-	59.7 ± 18.2	57.6 ± 12.3	-	-	-	-
Asemi, 2015 [46]	27	2.96± 2.84 **	2.0 ± 1.68 **	0.07	-	-	-	-	-	-	-	-	-
Moran, 2010 [47]	14	9.0 ± 6.0 **	7.10 ± 5.0 **	0.003	-	-	-	-	-	-	-	-	-
Olszanecka-Glinianowicz, 2008 [48]	15	-	-		6.0 ± 2.0	4.7 ± 2.1	>0.05	6.6 ± 3.0	6.1 ± 3.6	>0.05	-	-	-
Moran, 2007 [49]	12	5.50 ± 3.10 **	5.90 ± 3.30 **	0.066	-	-	-	-	-	-	-	-	-

Data presented as mean ± sd; * mg/mL; ** mg/L, RCT: Randomised control trial, ◊◊: The standard deviation after the intervention was calculated using a formula recommended by the Cochrane Handbook and validated in previous published study to determine the post-intervention standard deviation [50,51].

**Table 4:** Changes in circulating androgens and LH after diet-induced weight loss in PCOS women.

First Author, Year	*n*	Testosterone			SHBG (nmol/L)			DHEAS			Androstenedione			LH (IU/L)		
		Before	After	*p*	Before	After	*p*	Before	After	*p*	Before	After	*p*	Before	After	*p*
Mehrabani, 2012 [31]	26	-	-	-	26.9 ± 3.8 ^π^	37.6± 4.6 ^π^	<0.05	330.1 ± 30.6 *	298.1 ± 28.9 *	<0.01	-	-	-	-	-	-
Cheshmeh, 2021 [32]	99	1.35 ± 0.21 *	1.35 ± 0.25 *	0.96	33.12 ± 10.17	34.79 ± 10.59	0.25	363.39 ± 76.0 ^§^	383.06 ± 48.7 ^§^	0.1	1.97 ± 0.3 *	1.85 ± 0.2 *	0.01	5.94 ± 2.28	5.95 ± 1.8	0.77
Marsh, 2010 [33]	50	2.7 ± 0.2 ^π^	ND	<0.05	33.0 ± 2.9 ^π^	ND	<0.05	-	-	-	-	-	-	8.7 ± 1.1	5.3 ± 5.2 ◊◊	-
Esfahanian, 2012 [34]	13	0.83 ± 0.23	0.66 ± 0.21	<0.05	-	-	-	307.7 ± 358.5	293 ± 84.4	-	-	-	-	-	-	-
Deshmukh, 2023 [44]	11	1.5 ± 0.70 ^π^	1.39 ± 0.65 ^π^	0.11	16.0 ± 6.5 ^π^	22.8 ± 7.7 ^π^	0.002	6.9 ± 3.3 ^¥^	7.8 ± 3.4 ^¥^	0.4	5.3 ± 2 ^π^	4.7 ± 1.7 ^π^	0.3	8.2 ± 4.3	8.5 ± 4.1	0.09
Szczuko, 2018 [45]	22	0.57 ± 0.28 *	0.46 ± 0.11 *	<0.05	39.39 ± 15.94	44.34 ± 21.29	-	227.78 ± 66.8 ^δ^	228.41 ± 74.4 ^δ^	-	4.56 ± 1.6 *	3.90 ± 1.4 *	<0.05	7.07 ± 1.84	4.97 ± 1.5	<0.05
Olszanecka-Glinianowicz, 2008 [48]	15	2.4 ± 0.9 ^π^	2.0 ± 0.8 ^π^	-	30.2 ± 17.1 ^π^	33.2 ± 17.1 ^π^	-	525.7 ± 168.1 ^δ^	244. 3 ± 125.4 ^δ^	-	4.6 ± 2.0 *	5.4 ± 2.4 *	-	7.7 ± 5.8	9.3 ± 7.6	-

Data are presented as mean ± SD; ND: Not Determined; Units of measurement: * ng/mL; ^§^ ng/dl; ^π^ nmol/L; ^δ^ µg/dL; ^¥^ μmol/L; ◊◊: The standard deviation after the intervention was calculated using a formula recommended by the Cochrane Handbook and validated in previous published study to determine the post-intervention standard deviation [50,51].
